# Associations between clinically diagnosed testicular hypofunction and systemic lupus erythematosus: a record linkage study

**DOI:** 10.1007/s10067-017-3873-5

**Published:** 2017-11-03

**Authors:** Julia Pakpoor, Raph Goldacre, Michael J. Goldacre

**Affiliations:** Unit of Health-Care Epidemiology, Nuffield Department of Population Health, Big Data Institute, University of Oxford, Oxford, OX3 7LF UK

**Keywords:** Autoimmune disease, Epidemiology, Systemic lupus erythematosus, Testicular hypofunction, Testosterone

## Abstract

Systemic lupus erythematosus (SLE) has a high female predominance with a 9:1 female-to-male sex ratio, but males have poorer clinical outcomes than females. Gonadal hormones may mediate gender differences in SLE, but their role in SLE remains largely uncharacterised. We aimed to investigate a potential association between testicular hypofunction (TH), as a proxy for low testosterone levels, and SLE in males. A retrospective cohort study was conducted by analysing linked English national Hospital Episode Statistics (HES) and mortality data from 1999 to 2011. We calculated rates for SLE following TH, and TH following SLE, stratified and standardised by age, calendar year of first recorded admission, region of residence, and quintile of patients’ Index of Deprivation score. The adjusted rate ratio (RR) of SLE following TH was 7.7 (95% confidence interval (95% CI) 2.5–18.1, *p* < 0.0001). The adjusted RR for TH following SLE was 6.5 (95% CI 2.1–15.1, *p* < 0.0001). The positive association between TH and SLE supports a hypothesis that low testosterone levels may influence the development of male SLE. Of clinical importance, it suggests that males with SLE are at increased risk of co-morbid TH (regardless of which precedes which) and this may warrant consideration in the management of patients.

## Introduction

The demographic profile of systemic lupus erythematosus (SLE) is an interesting, distinctive feature of the disease. SLE has a 9:1 female-to-male sex ratio and most commonly affects young females following puberty, a key period of gonadal hormonal alteration and modulation [1]. Despite its female predominance, males are typically affected more severely, are thought to experience accelerated disease-associated damage, and experience renal complications more often including lupus nephritis and renal failure which represent a significant source of morbidity and mortality [2–4]. Gonadal hormones may be important in mediating gender differences in SLE and may contribute to its likely complex multifactorial pathophysiology incorporating genetic, hormonal, and environmental factors. This notion is supported by animal models of autoimmune diseases suggesting a potential immune-modulatory role of testosterone [5]. In murine models of SLE, disease activity has been shown to be increased by oestrogen administration and decreased by androgen administration [6, 7]. Though contentious, some cross-sectional studies have suggested lower testosterone levels in both males and females with SLE [8–10]. Any potential association between SLE and gonadal hormones remains poorly characterised. We aimed to investigate a potential association between testicular hypofunction (TH), as a proxy for low testosterone levels, and SLE in males using a linked dataset of English national hospital records.

## Methods

### Population and data

A national English record linkage dataset of Hospital Episode Statistics (HES) and mortality data from January 1999 to December 2011 was used to undertake a retrospective cohort study. HES incorporate every episode of hospital day-case or overnight inpatient care in National Health Service (NHS) hospitals [11]. The Oxford record linkage group was responsible for undertaking the record linkage and thereby constructing a time-sequenced record of successive episodes of care (and death, if it took place) for each person [12]. Approval for the use of the datasets was provided by the Central and South Bristol Research Ethics Committee (ref 04/Q2006/176).

A cohort of males with TH (ICD10 code E29.1) was created through identification of the earliest known recorded episode of day-case care or hospital admission in which TH was coded in males within the study period. A similarly constructed reference cohort identified the earliest known admission for each male admitted for a range of mainly minor medical and surgical conditions.^1^ A wide range of conditions were selected in accordance with standard epidemiological practice [13]. Anyone with a record of SLE prior to, or at the same time as, the admission for TH or reference condition was excluded from the cohort.

The cohorts were searched for any subsequent day-case or inpatient record for, or death from, SLE (ICD10 code M32). We considered that the rates of occurrence of SLE in our reference cohort would reflect those of the general population while allowing for migration in and out of it (migration information was not obtained).

In considering the possibility of reverse causality, using the same methodology and reference cohort, we similarly constructed a cohort of males with SLE and searched for subsequent day-case or inpatient care for TH. A concurrent or previous admission for TH before SLE was excluded, thereby ensuring that in considering both analyses (TH before SLE and SLE before TH) no individual was counted twice. In all analyses, records of TH and SLE were included whether the diagnoses were recorded as the main or as a subsidiary diagnosis.

### Statistical methods

Date of entry into each cohort was the date of the first admission for TH, or reference condition, and date of exit was the date of the first record of SLE, death or the end of data collection (December 2011). We calculated rates of SLE based on person-days at risk. We stratified and then standardised the cohorts by age (in 5-year age groups), calendar year of first recorded admission, region of residence, and quintile of patients’ Index of Deprivation score (a standard English measure of socio-economic status). The methods used in the calculation of observed and expected numbers in our disease association studies have been published in detail elsewhere [13, 14]. The indirect method of standardisation was used, taking the combined population of the TH and reference cohorts as the standard population. We multiplied the stratum-specific rates in the standard population by the number of person-days in each stratum of the TH cohort and then, separately, the reference cohort, to obtain the expected number of people with SLE in each stratum of each cohort. Expected numbers were then summed across all strata to give expected totals for each cohort, which were then compared with the observed totals. Rate ratios (RR) were calculated by taking the standardised rate of occurrence of SLE in the TH cohort relative to the reference cohort, mathematically equivalent to the formula (O^TH^/E^TH^)/(O^REF^/E^REF^), where O and E are the observed and expected numbers of SLE cases in the TH and reference cohorts, respectively. The confidence interval for the RR of SLE and *χ*2 statistics for its significance were calculated as described elsewhere [15].

## Results

There were 5045 males in the TH cohort and 4.4 million in the reference cohort. The adjusted RR of SLE following TH was 7.7 (95% confidence interval (95% CI) 2.5–18.1, *p* < 0.0001), based on 5 cases observed, and 0.7 expected. The adjusted RR of TH following SLE was 6.5 (95% CI 2.1–15.1, *p* < 0.0001), based on 5 cases observed, and 0.8 expected, from a total of 3473 males in the SLE cohort. None of the 10 cases with both TH and SLE had a diagnosis of Klinefelter syndrome in any diagnostic position on the same record as the record for TH.

## Discussion

The strong positive association between TH and SLE gives some support to a hypothesis that low testosterone levels may influence the development of male SLE. However, we cannot be sure about the direction of cause. Of clinical importance, the study suggests that males with SLE are at an increased risk of co-morbid TH (regardless of which precedes which) and this may warrant consideration in the management of patients. Patients with TH who are seen in hospital may be at the more severe end of the spectrum of the condition, and an association between low testosterone and SLE may be more prevalent at a primary care or subclinical level. A key strength of this study is its use of a very large dataset. Even with a dataset this size, there were just ten males with co-morbid TH and SLE. Datasets in smaller populations covered by record linkage, or with other types of electronic medical records, may not have the power to detect such an association. We considered, as potential confounders, the factors used in standardisation (see the ‘Methods’ section), but we cannot exclude the possibility that other confounding factors, or that some other aspects of TH, instead of low testosterone levels, may underlie the association; or that SLE and TH may share currently unknown risk factors.

Limitations include that this study is not a follow-up study of a cohort from the point of initial diagnosis, but rather, uses prevalent cases of TH based on the first recorded hospital-based episode. We expect that the majority of individuals with SLE will have been seen as a day-case or inpatient at least once, but are unable to confirm this. Further, information about individuals who emigrate or receive treatment outside the included geographical region was not obtained.

Our results support a growing evidence base suggesting a role for low testosterone in the risk and/or clinical course of autoimmune diseases in males. For example, a population-based nested case-control study has shown that lower levels of testosterone are predictive of RF-negative rheumatoid arthritis (RA), indicating that low testosterone levels precede RA onset and influence disease phenotype in males [16]. Male multiple sclerosis (MS) patients have a high prevalence of hypogonadism which is also associated with poorer cognitive and clinical outcomes, and a strong association (a fivefold elevation of rates) has previously been suggested between TH and subsequent MS risk [17–19]. Further, males born with Klinefelter syndrome (characterised by the presence of an extra X chromosome (XXY) and consequently lower testosterone levels) are at increased risk of numerous autoimmune diseases, including SLE [20]. This leads us to speculate that the observed association between TH and SLE is unlikely only to be a consequence of the disease, but rather a reflection of a role for testosterone in the development and/or disease modulation of autoimmune diseases more generally; this is in line with proposed anti-inflammatory properties of testosterone in suppression of the cellular and humoral components of the immune system [5]. Our findings, though far from definitive, are probably novel in that a literature search for ‘testicular hypofunction’ and ‘systemic lupus erythematosus’ did not yield any publications.

In conclusion, we show an association between clinically diagnosed low levels of testosterone and SLE using a large national population-based dataset. We hope to stimulate future studies measuring testosterone levels directly in both males and females in determining direction of causality, and the exploration of potential interactions between testosterone and other postulated SLE risk factors; and, in the meanwhile, to encourage clinicians to be aware of TH as a potential source of reduced disease-associated quality of life in these patients. Elucidating the role gonadal hormones play in gender differences of SLE may be important for the development of preventative strategies and gender-specific treatment approaches.

## Footnotes

1 Conditions used in reference cohort: cataract, otitis externa, otitis media, varicose veins, haemorrhoids, deflected nasal septum, nasal polyp, gall bladder disease, inguinal hernia, in-growing toenail and other diseases of nail, sebaceous cyst, internal derangement of knee, bunion, selected limb fractures, dislocations and sprains, superficial injuries and contusion, appendectomy, tonsillectomy, adenoidectomy, hip replacement, and knee replacement.
